# Patient and injection partner perspectives on barriers and facilitators to home-based administration of long-acting injectable antiretroviral therapy

**DOI:** 10.1371/journal.pone.0341173

**Published:** 2026-03-09

**Authors:** Alicia T. Bolton, Beth Bourdeau, Greg Rebchook, Jonathan Van Nuys, Erin Moore, Kate Buchacz, Jesse O’Shea, Parya Saberi

**Affiliations:** 1 Department of Medicine, University of California, San Francisco, San Francisco, California, United States of America; 2 Division of HIV Prevention, Centers for Disease Control and Prevention, Atlanta, GeorgiaUnited States of America; Addis Ababa University, College of Health Sciences, ETHIOPIA

## Abstract

Long-acting injectable antiretroviral therapy (LAI-ART) offers an alternative to daily oral treatment but is typically administered in clinics, which can create barriers for some people with HIV (PWH). Home-based administration by trained treatment buddies (TBYs)—trusted partners, friends, or family members—has not been systematically studied. We conducted semi-structured interviews with 31 participants (16 PWH and 15 TBYs) across 4 HIV clinics in the San Francisco Bay Area between June 2024 and April 2025. Guided by the Consolidated Framework for Implementation Research (CFIR), interviews explored anticipated facilitators, barriers, and training needs for home-based LAI-ART. Participants identified several anticipated benefits of home-based LAI-ART, including increased convenience, reduced transportation burdens, enhanced privacy, comforting and emotionally supportive care, and opportunities to foster empowerment and shared responsibility between PWH and TBYs. Key barriers included concerns about medication storage and delivery logistics, maintaining reliable injection schedules, needlestick safety, and the readiness and confidence of TBYs. Participants emphasized the need for hands-on training, ongoing support, and clear protocols to ensure safe, acceptable, and effective home-based administration. These findings underscore the importance of proactive planning and tailored support in addressing both the technical and emotional dimensions of home-based LAI-ART. Anticipating these needs can facilitate the successful implementation and expand access to person-centered HIV care.

## Introduction

In 2022, the United States released the National HIV/AIDS Strategy [1], which aimed to promote therapies that achieve sustained viral suppression and maximize the uptake/adoption of those therapies to address the remaining 35% of persons with HIV (PWH) who were not yet virally suppressed [2]. Long-acting injectable (LAI) antiretroviral therapy (ART) is a promising, effective strategy [3] that is preferred by many PWH [4–9], due to eliminating the need for daily oral ART and improving their quality of life [10–12]. Studies have shown that simplifying ART regimens can improve health outcomes [13].

However, the adoption of LAI-ART faces several challenges for scale-up [14], including increased clinic visits [9,15,16], concerns about privacy, and stigma experienced in medical settings [17,18]. The need for ongoing clinic injection appointments can be financially and logistically prohibitive for some PWH, which can widen existing healthcare disparities, particularly among individuals facing multiple structural barriers to accessing care [9,19–21]. Each cabotegravir/rilpivirine (CAB/RPV) visit is estimated to last 30 minutes to an hour (excluding travel time to and from the clinic) [17,22], which may not be feasible for some PWH and clinics.

To maximize the reach and impact of LAI-ART, alternative administration models that align with patient preferences must be developed. Studies have demonstrated that PWH are interested in options besides clinic administration, including home-based administration [6,23–25]. One pilot study has provided preliminary evidence that a home visit by a licensed nurse was a comparable and acceptable alternative to clinic-based LAI-ART administration [26]. Additional research indicates a strong interest among PWH in home-based LAI-ART administered by a trusted partner, friend, or family member [4,23], which aligns with their preferences for privacy, convenience, autonomy, and increased self-efficacy in managing their health [5,6,17,27].

In this paper, we focus on the feasibility and acceptability of providing LAI-ART to PWH at home by a trained person of their choice, as part of the Innovative Administration of Long-Acting Injectables for HIV Treatment Enhancement at Home (INVITE-Home) study [28]. INVITE-Home is the first study to examine the implementation of home-based LAI-ART administered by a trained “treatment buddy” (TBY), such as a family member, partner, or friend. This formative research gathered input from PWH and individuals whom they identified as potential TBYs to be trained to administer LAI-ART injections, with the goal of providing information for a home-based LAI-ART injection training curriculum grounded in the needs and concerns of PWH and TBYs.

## Materials and Methods

### Study setting and design

This qualitative study was conducted between June 2024 and April 2025 across 4 HIV clinics in the San Francisco Bay Area: [**1]** Ward 86 at the San Francisco General Hospital, a public Ryan White safety-net clinic; **[2]** the University of California, San Francisco's (UCSF's) Women's HIV Program, which provides trauma-informed care for women with HIV; **[3]** UCSF's 360 Wellness Center, which primarily serves African American men and adults aged 50 and older with HIV; and **[4]** the Adult Immunology Clinic at Highland Hospital, which delivers HIV care in the East Bay. One-on-one, semi-structured interviews were conducted with PWH and their candidate TBYs to explore potential barriers and facilitators to home-based LAI-ART.

### Participants

PWH were eligible if they were ≥18 years of age, receiving care at a partnering clinic, were receiving CAB/RPV for HIV treatment, and could identify a TBY to whom the PWH had already disclosed their own HIV status and who would be willing to administer injections. All PWH had received at least one CAB/RPV injection in the clinic prior to their interview, none were excluded based on prior injection history. TBYs were eligible if they were ≥18 years of age, identified by a participating PWH, and willing to consider providing LAI-ART injections.

### Recruitment and informed consent

Clinic staff notification and advertisement via posted study flyers facilitated recruitment. PWH contacted the study team and completed a telephone screening for eligibility. Eligible PWH provided contact information for a candidate TBY. Interest in home-based injections was established during recruitment and screening, prior to the interviews. This study was approved by the UCSF Institutional Review Board (IRB). A study team member obtained and documented verbal informed consent in accordance with IRB approval from all PWH and TBYs prior to participation using an IRB-approved information sheet. This information sheet summarized the study purpose, procedures, and interview topics but did not include detailed instructions on LAI-ART procedures, injection technique, needlestick risk, or access to emergency PEP. Thus, participants were not pre-briefed on these specifics before data collection. However, certain safety topics (e.g., needlestick risk) were explicitly probed in the interview guide, which may have prompted some reflections. All interviews took place before the following protocol phase, which involved the administration of home-based injections (see invitehome.ucsf.edu).

### Data collection

Parallel semi-structured interview guides were developed for PWH and TBYs to explore knowledge of and experiences with LAI-ART, attitudes toward home administration, perceived facilitators and barriers, TBY selection, and training and support needs. Interviews were conducted separately to center each participant’s perspective. Because interviews were conducted before any injections, they focused on anticipated experiences and preferences. The first author (ATB), with support from the study clinician (JVN), conducted all interviews virtually [29] via a Health Insurance Portability and Accountability Act (HIPAA)-compliant Zoom platform. Interviews were audio-recorded and lasted 45–60 minutes. Participants received a $40 (US) e-gift card.

Field notes were taken during and after each interview to capture participant perspectives and contextual insights not evident in the transcripts. Audio recordings were transcribed using Zoom’s automated transcription feature, reviewed for accuracy, and de-identified by study staff. Each participant was assigned a unique identifier.

### Analysis

Data collection and analysis occurred concurrently and continued until thematic saturation was achieved. Transcripts were uploaded to Dedoose [30], a web-based qualitative data analysis platform, and thematically coded using a hybrid inductive-deductive approach. The Consolidated Framework for Implementation Research (CFIR 2.0) [31,32] guided analysis across five domains:

- Innovation: Attributes of home-based LAI-ART (e.g., adaptability, complexity, relative advantage, and design quality) as perceived by PWH and TBYs.
- Inner setting: The environment in which the intervention is delivered, its physical and social characteristics, and the overall household context that shapes implementation.
- Outer setting: The broader external context influencing implementation, such as healthcare policies, reimbursement structures, external partnerships (e.g., referring clinics or pharmacies), and sociocultural conditions that affect access, engagement, and continuity of care.
- Characteristics of individuals: Attributes of those involved in home-based LAI-ART, primarily PWH and their TBYs, including their beliefs, knowledge, motivation, confidence, self-efficacy, and readiness to engage in or support home-based treatment.
- Implementation process: The strategies and activities used to plan, deliver, and refine implementation, including coordination, development of communication tools, workflow adaptations, and continuous reflection and quality improvement.

A preliminary codebook was developed by the primary analyst (ATB) and refined with the study team (ATB, BB, GR, PS). Manual coding in Dedoose was supplemented with AI-assisted coding via UCSF’s HIPAA-compliant Versa platform, which uses OpenAI’s GPT-4o model [33], to enhance analytic rigor. All AI-generated coding outputs were reviewed by the primary analyst (ATB). When discrepancies arose between manual and AI-assisted coding, the analyst re-examined the relevant excerpts in Dedoose, consulting with the study team as needed, and adjusted codes accordingly. This iterative process of cross-checking and refinement served to strengthen the credibility and completeness of the analysis, without privileging AI-generated codes [34]. Final codes were mapped to CFIR domains, and illustrative quotes were selected to highlight key themes.

## Results

We conducted interviews with 31 participants (16 PWH and 15 TBYs), resulting in 15 complete dyads and 1 individual PWH whose TBY could not be reached: 12 individuals from UCSF's 360 Wellness Center; 8 from UCSF's Women's HIV Program; 7 from San Francisco General Hospital's Ward 86 (with 1 unreachable TBY); and 2 from the Adult Immunology Clinic at Highland Hospital. Table 1 summarizes PWH and TBY characteristics.

**Table 1:** Participant characteristics (N = 28*).

Characteristic	PWH (N = 16)	TBY (N = 12)
**Age, mean years (SD)**	54.9 (10.0)	50.3 (10.0)
Race/ethnicity**, N (%)		
Asian	1 (6)	1 (8)
Black/African American	6 (38)	4 (33)
Hispanic/Latino	2 (13)	2 (17)
White	7 (44)	3 (25)
Multiracial	0 (0)	2 (17)
Sex, N (%)		
Female	6 (38)	5 (42)
Male	10 (63)	7 (58)
Sexual orientation, N (%)		
Gay or lesbian	8 (50)	4 (33)
Bisexual/pansexual	1 (6)	0 (0)
Heterosexual or straight	7 (44)	8 (67)
Highest level of education completed, N (%)		
Less than a high school diploma	4 (25)	1 (8)
High school graduate/GED	3 (19)	2 (17)
Some college	5 (31)	4 (33)
College graduate or above	4 (25)	5 (42)
Relationship type**, N (%)		
Significant other (e.g., partner, spouse, boyfriend/girlfriend)	6 (38)	5 (42)
Family member (e.g., parent, sibling, aunt/uncle, cousin)	3 (19)	3 (25)
Friend	6 (38)	2 (17)
Roommate	2 (13)	2 (17)
Neighbor	1 (6)	1 (8)
Caregiver	0 (0)	1 (8)

* Data available for 28 of 31 participants.

** Participants allowed more than 1 response option. Multiracial indicates selection of more than 1 race/ethnicity; all other categories reflect a single response. Hispanic/Latino was selected as a single category.

Interview findings delineated anticipated facilitators and barriers to home-based LAI-ART from PWH and TBY perspectives (Table 2). Exemplary quotes related to each facilitator are listed in Table 3, quotes pertaining to barriers are noted in Table 4, and quotes related to themes noted as facilitators and barriers are in Table 5.

**Table 2:** Anticipated facilitators and barriers to home-based LAI-ART implementation by CFIR domains.

CFIR domain	Facilitator	Barrier
**Innovation**	• Convenience and access • Comprehensive training, education, and ongoing support • Protecting privacy and reducing stigma • Comforting and emotionally supportive care • Fostering autonomy and mutual empowerment	• Proper injection technique and administration • Needlestick injury risk and associated safety concerns for TBYs • Ensuring appropriate medication storage and handling • Maintaining timely and reliable injection schedules
**Inner setting**	• Medication delivery*	
	• Ongoing communication and follow-up	• Access to essential injection supplies and safe disposal options • Preparing a safe and distraction-free home injection environment
**Outer setting**	• Laboratory monitoring and coordination*	
	—	• Insurance coverage and cost considerations • Potential loss of clinic connection and routine
**Characteristics of individuals**	• TBY selection considerations* • Relationship dynamics that support or challenge care*	
	—	• Injection anxiety and emotional readiness
**Implementation process**	—	—

CFIR, Consolidated Framework for Implementation Research; LAI-ART, long-acting injectable antiretroviral therapy; TBY: treatment buddy. “—” indicates no specific facilitators or barriers were identified for this domain.

* Indicates both facilitator and barrier

**Table 3:** Exemplary quotes from participants related to facilitators associated with home-based LAI-ART.

Facilitator	Participant demographics	Quote
Convenience and access	PWH01: 46-year-old, Black/African American, female	“I have rheumatoid arthritis and heart problems, and so sometimes it’s a struggle to get to the doctor… That’s the main reason why I would love it to be at home, because when I have days like that, I don’t have to worry about how I’m going to get there.”
	PWH02: 53-year-old, White, male	“I wouldn’t have to drive to [clinic] every other month… It took me two hours and 15 minutes yesterday to get to [clinic] for my eight o’clock appointment. I left at 5:45 am, so I got there with five minutes to spare.”
	PWH03: 36-year-old, Black/African American, male	“It comes down to convenience and accessibility. Less time in the car, less money on gas… less worry dealing with scheduling… it comes in the mail, you wake up, take care of it, and get about your day… that’s huge. The convenience for me is the biggest piece of the puzzle.”
	TBY02: 56-year-old, Multiracial, female	“It’s more freedom for [PWH]… For him to not go so far driving is a big thing, and from [where we live] it takes a while. And it benefits us, too, that he is home when my mom, our mom, is here. She’s 93 years old, so it would help that there’s always someone here with her… When he has his appointments, he leaves really early because of traffic time, and then coming home takes almost all day for him... Not to mention toll, and he’s on a fixed income. So, it’s a lot of concerns. This is exciting to have him do this at home.”
	TBY06: 53-year-old, Black/African American, male	“When she goes to her monthly appointments, that’s an entire day. It takes up a lot of time... Being able to do it in the home will save the stress and the cost of getting to the [clinic]… Having it done right here in the home is definitely beneficial.”
	PWH04: 62-year-old, White, male	“I live in San Francisco, 10 min to [clinic], so for me it’s probably easier just to go to the doctor’s office than have it delivered, refrigerated, call my friend, ‘Hey, it’s here. Can you come now?’ I think the time period would actually be longer and more complicated…Inconveniencing somebody for my benefit, because they would have to go through training, and then every other month, I would have to ask them to be here at a specific time.”
Comprehensive training, education, and ongoing support	PWH05: 65-year-old, Hispanic/Latino, male	“I have the confidence that you guys are gonna educate her in the right way, so then she can do it to me.”
	PWH06: 55-year-old, White, female	“He knows me the most out of anybody… As long as he’s trained and he’s comfortable, then I’m comfortable.”
	PWH04: 62-year-old, White, male	“I would want to make sure that the person is trained properly, and feels very comfortable doing it… I know if it’s not given in the right spot, you could actually do some damage. So, I would want to make sure that that person really understands where exactly it needs to be given.”
	TBY04: 57-year-old, Hispanic/Latina, female	“If I’m trained, I would feel absolutely comfortable doing it.”
	TBY05: 38-year-old, Hispanic/Latina, female	“I’m willing to do it, of course, with the right training. That was my fear. I was like, what if I did it wrong? So, the training part would be important, but I would be willing to do it.”
	TBY04: 57-year-old, Hispanic/Latina, female	“You’re providing the training, you’re providing support, and [the study clinician] is there, I think all of those would make me feel comfortable.”
Protecting privacy and reducing stigma	PWH02: 53-year-old, White, male	“It would be more comfortable getting shots at home because you are not in the medical office where everyone’s there, and they see you, except behind a curtain.”
	PWH07: 62-year-old, Black/African, American, male	“These people [at the clinic] live in your community, and you don’t know what’s being said… When you can just have the medicine come to your home, and your spouse or your family member knows how to do the injection through the training, that makes it all even better, you know, respectfully better.”
	PWH08: 48-year-old, Hispanic/Latino, male	“When I have to explain myself to someone, ‘Oh, I have to go to the hospital tomorrow,’ I don’t like letting them know I’m going to the hospital because then they ask you, ‘What’s wrong?’, or ‘Are you okay?’ I don’t like that.”
Comforting and emotionally supportive care	PWH05: 65-year-old, Hispanic/Latino, male	“It’s just more comfortable at home.”
	PWH09: 64-year-old, Black/African American female	“Going to the clinic is nice, but to do it at home is like more comfort. You’re more in your comfort zone, and you can be more relaxed.”
	PWH10: 61-year-old, White, male	“I love seeing [provider], but… I think often patients feel better at home. And this would be just kind of a continuation of that general philosophy.”
	TBY08: 41-year-old, White, male	There’s just something to being at home in your safe space that just kind of puts somebody at ease… I think even if it was like a nurse going to their home and doing it, I think that alone would change the way they felt. It’s not in your typical medical environment… Some people in a medical area get uneasy by it… So, I could see why being at home you’ll be nice and relaxed, and also your buddy is doing it. So, you’re gonna be trustworthy of them and comfortable.”
	TBY01: 51-year-old, Black/African American, female	“At home it’s a lot quieter than in the clinic where we have a full staff, and we’re dealing with a lot of clients and nurses and providers all together in this one big room. I think it will be a lot simpler, a lot safer and easier for her… It’s in the privacy of her own room, her home... If it does cause her any irritation or irritableness, she’s right here. She’s at home. She doesn’t have to travel to and from the clinic site. I’ll be here to help if she needs any help.”
Fostering autonomy and mutual empowerment	PWH10: 61-year-old, White, male	“The things that motivate me are convenience, autonomy, a sense of taking control of my own healthcare. There’s lots of studies that show that when patients are educated and participate in their healthcare, that their outcomes are better. My prediction is that people are going to have more consistently undetectable [HIV viral load test] results.”
	PWH10: 61-year-old, White, male	“It would be empowering. They’re learning a new skill that they would be competent in. I think that there’s a wellness component in that. Doing something for someone else is gratifying. It gives a person a sense of purpose, a sense of doing something that’s of significant value.”
	PWH07: 62-year-old, Black/African American, male	“She’s already a very caring person, a compassionate person, so that would give her more strength, and just the educational part will boost her morale, and she’ll be great at it… And we can get a lot from that. And then we can sit down… we can have these conversations as to how this works, and how that works, and empower each other with it.”
	TBY09: 63-year-old, Black/African American, male	“I want to learn to be able to take care of her, to do that for her... And me having that experience that you’re gonna teach me, it’s gonna improve me, so I feel good about it… It’s something I really want to learn. That’s the best part about it; I’m getting two pluses: I’m getting some knowledge, and I’m getting a chance to take care of and do something for my wife.”
	TBY02: 56-year-old, Multiracial, female	“This is my living, this is my life, this is my job, my career, and my passion.”
	TBY07: 54-year-old, Black/African American, female	“It’ll give me the education about it, which could possibly lead me to help others… It might help me… just the fact that [study clinician] is teaching me how to do this will really make me happy, because I’ll have something to do and look forward to doing. And I really would love to go out and help do stuff to help other people.”
Ongoing communication and follow-up	PWH05: 65-year-old, Hispanic/Latino, male	As one PWH explained: “It’s just a matter of being able to talk to somebody when you need to at any point to check in with. Maybe [clinician trainer] could check in with [PWH] at some point after three or four injections.”
	PWH06: 55-year-old, White, female	“Just having a good relationship with you guys, so if I have any concerns or [PWH] has any, we can call you or video you.”
	PWH03: 36-year-old, Black/African American, male	“It’s a lot of lag time for some people in between the two [injections]... Maybe having a reminder sent out to people.”
	TBY11: 64-year-old, Asian, male	“There could be just a general understanding that after each injection, we get on MyChart and let our provider know that we got the injection…”

**Table 4:** Exemplary quotes from participants related to barriers associated with home-based LAI-ART.

Barrier	Participant demographics	Quote
Proper injection technique and administration	PWH04: 62-year-old, White, male	“I know if it’s not given in the right spot, because once I had issues where maybe they had hit a nerve or something, where for several days I had some problems in my legs. So maybe somebody hit something… Going to the wrong point, you could actually do some damage.”
	TBY11: 64-year-old, Asian, male	“It’s a matter of finding the correct location and how to deliver the medication. Do you push it slowly? How slow? And do you rub the site afterwards? So, a little demonstration would be helpful.”
	TBY06: 53-year-old, Black/African American, male	“I’ve never done that before, so I wanna make sure it’s done absolutely correctly.”
Needlestick injury risk and associated safety concerns for TBYs	PWH10: 62-year-old, White, male	“Obviously, personal protective equipment and all of that and the treatment buddy understanding the risks of transmission if they were to get stuck… and what the protocol would be if they did get stuck, and the importance of timing... if there was a needlestick, having an emergency post-exposure prophylaxis as part of the equipment that people have at their homes.”
	TBY03: 40-year-old, White, male	“…what would be the protocol after that incident occurs? Do I have to dispose of the needle, and if I do, do I have a backup?... I don’t know if I should be concerned about any of the medication exiting the tip of the needle and entering my system. I don’t know if that would be something to be concerned about or not.”
	TBY12: age not reported, White, male	“Yeah, Hep C, or something like that, would concern me.”
	TBY08: 41-year-old, White, male	“If an accident was to happen, I know that I have options… time is of the essence... so I would have to immediately get to an emergency room and tell them what happened just to be safe, because I’m not positive, and that’s not something I want to be… I’d like to stay not positive.”
Ensuring appropriate medication storage and handling	PWH08: 48-year-old, Hispanic/Latino, male	“Would it go bad if it stays [out] more than the required time to be refrigerated? Let’s say it stays out in the post office or something, and they bring it late.”
	TBY11: 64-year-old, Asian, male	“I understand the medication is sent to your house, and it has to be refrigerated, so you have to be there to receive it. I don’t know how long it can sit in a cold box in front of your house.”
	TBY07: 54-year-old, Black/African American, female	“Is it possible it’s gonna go bad? What’s the temperature it has to be at all times? [What] if we have a power outage?”
	PWH10: 61-year-old, White, male	“The refrigeration might be an issue in terms of maintaining the appropriate temperature so there’s not any denaturing of compounds within the medication.”
	TBY13: age not reported, race/ethnicity not reported, male	“I know that when you do it, the needle has pressure. So, you have to draw it out slowly, and it like, wants to go back into the suction… so just the process of like drawing it all out to the right amount and not wasting any.”
	TBY03: 40-year-old, White, male	“What if somehow, I mess up introducing the drug into the needle? What’s my backup? I assume that’s not an emergency, but I would need to get access to another vial somehow.”
Maintaining timely and reliable injection schedules	PWH14: 58-year-old, Black/African American, female	“I just want them [TBY] to know what’s really important… that it has to be done that day instead of saying like, ‘I’ll do it tomorrow’ or something like that.”
	PWH08: 48-year-old, Hispanic/Latino, male	“I just think about doing it on the exact date, on the required date. I don’t wanna forget.”
	TBY11: 64-year-old, Asian, male	“Maybe planning out the schedule for the full year. Then we could just mark it on our calendar, and we know it’s coming up, we know when we need to get our blood drawn. That helps us plan vacations.”
	TBY10: 33-year-old, Multiracial, male	“I guess just knowing that you have a window of time is helpful to know, but you also want to emphasize how important it is to get it as close to the 4-week mark as possible or that 8 weeks, and then what the repercussions are if you don’t.”
Access to essential injection supplies and safe disposal options	PWH10: 61-year-old, White, male	A PWH noted: “Disposing of sharps would be a big issue. And I know that Walgreens, for example, is no longer giving out sharps containers.”
	TBY02: 56-year-old, Multiracial, female	“We have grandkids and animals, so I wanna make sure that we dispose of it quickly, it’s not laying around.”
Preparing a safe and distraction-free home injection environment	PWH06: 55-year-old, White, female	“We’d have to shut the dogs out ‘cause they’d be all over us, so we’ll put him in another room… maybe have a sheet like you guys have at the hospital… to lay down a sheet first, because they’re always on the bed.”
	PWH06: 55-year-old, White, female	“My bed is kind of low to the floor. You’d have to be crouched down almost on your knees to be able to do it.”
	PWH04: 62-year-old, White, male	“They don’t want somebody to be crouched over, so finding the right space in the house… And for the person who’s giving the injection, that they don’t end up with a back issue as a result of it.”
	TBY12: age not reported, White, male	“…somewhere in the person’s home that’s just clean and organized”
Insurance coverage and cost considerations	PWH11: 72-year-old, Asian, male	“[Clinician] put in a grant for me that would cover my injections. Is that gonna be the case when I’m at home? Is there gonna be shipping and delivery charges? Is it gonna be more costly?... I just wanna make sure that I don’t have to pay from my pocket.”
	PWH09: 63-year-old, Black/African American, female	“If I have to pay for it, then that would be hard on me.”
Potential loss of clinic connection and routine	PWH01: 46-year-old, Black/African American, female	“It might not be a barrier, but it is a disadvantage, because maybe the person only gets out of the house to go to the doctor.”
	PWH12: 59-year-old, White, male	“I see my doctor first, and then I get my injection. So, I’m killing two birds with one stone. I’m not really there just to receive my shot… That is a positive, because I really am seeing my doctor now every two months, but that will probably end.”
Injection anxiety and emotional readiness	PWH16: 43-year-old, Black/African American, female	“It might be a little scary cause does she really know what she doing, or is she heavy handed? When she sticks me, is it gonna hurt? And then, is she gonna be nervous?”
	TBY09: 63-year-old, Black/African American, male	“I’m concerned about hurting her when I poke her... That is my number one concern.”
	TBY06: 53-year-old, Black/African American, male	“I never wanna put myself in a position where I’m harming my partner, my wife… If I ever felt like I was doing any harm, or I hurt [her], or made a mistake, and maybe inject her in the wrong way, I probably wouldn’t wanna do it again.”
	TBY08: 41-year-old, White, male	“If I actually administer it improperly, like in the wrong area, or if for some reason the device malfunctioned or something, and for whatever reason it caused him pain… or swelling, or something.”
	TBY12: age not reported, White, male	“I’d be concerned about skin infections or something deeper than that, you know, cleaning the area properly and stuff, and getting the injection to the right area.”
	PWH12: 59-year-old, White, male	“I don’t like needles… I don’t like getting shots, and I have a tendency to tense up when I’m getting shot in the rear… so I’m a little apprehensive about having somebody who is a non-professional do this.”
	PWH12: 59-year-old, White, male	“[My TBY] said he also has a hard time with needles, so whether or not he could do them for me, that’s gonna be tough to find the right person… comfortable around needles. I mean, you kind of have to be that, and not squeamish.”
	TBY06: 53-year-old, Black/African American, male	“That’s gonna be a big challenge for me. I scream sometimes when I’m at the dentist getting my checkups… I’m gonna have this here to somebody I love, and I mean me personally, if anything comes up, any doubt, if I don’t feel right, I won’t do it. You know what I mean, like I won’t. Hopefully, whatever training is gonna help out a lot to make sure I know what I’m doing. And then she has to be comfortable also, you know.”
	TBY05: 38-year-old, Hispanic/Latina, female	“The thought of putting a needle is kinda scary, but I think once I get past it the first time, I’d be fine… I think I could do it. It’s just the thought of it at first is kind of a little weird.”

**Table 5:** Exemplary quotes from participants related to facilitators and barriers associated with home-based LAI-ART.

Facilitator and barrier	Participant demographics	Quote
Medication delivery	PWH15: 48-year-old, White, female	“I have my other meds delivered too to my house... It comes at the same time every month. I already sign for my meds… It saves me a trip to the hospital every month… It works better that they come in the mail, and then I have them in a box here already.”
	TBY03: 40-year-old, White, male	“…with UPS, they notify me when items are getting delivered in specific times, so having the tracking numbers, I think, is pretty important, especially for something that has to be signed.”
	PWH03: 36-year-old, Black/African American, male	“If it is being mailed to the home, is it in a timely manner so that you’re ordering it ahead of time, early enough to ensure that it’s coming at the appropriate time to give it within the correct 7-day window?”
	PWH12: 59-year-old, White, male	“…I have to deal with shipping... That’s always a concern, too, because packages get stolen. I’m in an apartment complex, and they have recently, because of theft of packages, started locking up our packages for us…”
	PWH12: 59-year-old, White, male	“I would like consistency on when the packages are going to be delivered…”
	PWH03: 36-year-old, Black/African American, male	“For the single person who’s not home when they’re working and they’re commuting and they get home at some funky hour, it’s like making sure that it gets delivered at a time that they can actually sign to get it…”
Laboratory monitoring and coordination	PWH04: 62-year-old, White, male	“Just thinking about the convenience, I always tie my lab work into the doctor visits. If you do the lab work, the convenience factor is gone. Yeah, then I’d have to go and do the lab work.”
	PWH04: 62-year-old, White, male	“I only do lab work twice a year. And I was thinking about that because I could sit there wonder if [TBY] did this right? Maybe I might feel more comfortable to do lab work more frequently during the in-home administration. Every other month, there’s only six, so maybe do it at least three or four times a year to make sure that you stay undetectable, that it was given the appropriate way.”
	TBY12: age not reported, White, male	“I’d like to know the person’s viral load is still zero, that I’m administering it in the right area or at the right depth.”
TBY selection considerations	PWH05: 65-year-old, Hispanic/Latino, male	“You don’t want them to show up on dope or drunk… They gotta have control of all that kind of stuff.”
	PWH10: 61-year-old, White, male	“…needs to understand the gravity of what they’re doing… It’s not like helping a friend lose weight by going on a walk with them. This could have significant implications.”
	PWH10: 61-year-old, White, male	“Someone the patient feels genuinely cares about their well-being and is consistent and unlikely to change,” one PWH explained, “because switching treatment buddies, you want to minimize that occurring.”
	PWH14: 58-year-old, Black/African American, female	“The longer you know a person, you know their quirks and everything. You don’t want somebody that you just met. You want somebody that you’ve known for a while, and that you have a good relationship with… [for] a good couple of years, like five, six years.”
	PWH16: 43-year-old, Black/African American, female	“Are you sure this person is going to be in your life for a year?”
	PWH13: 45-year-old, White, male	“It would be somebody that you’d normally see on a daily or weekly basis… somebody that you know, that’s around.”
	PWH08: 45-year-old, Hispanic/Latino, male	“They’re gonna inject me in my butt, so I need someone that I could trust with this part of my body.”
	TBY03: 40-year-old, White, male	As one asked, “If something unexpected happens, an emergency happens, which is life, life happens, what do I do? If it can’t happen on the scheduled date, if it’s for like a funeral or I have to go back to the East coast, do I travel with this?”
Relationship dynamics that support or challenge care	PWH06: 55-year-old, White, female	“It would probably bring us closer together even more as a couple, you know. He’s not [living with] HIV, so he can learn a little bit more about what’s going on with me, and I think it’s a great thing actually.”
	TBY07: 54-year-old, Black/African American, female	“It will motivate me to just help him with his injections, knowing he knows that even after 17 years, he can trust me, even though we know he knows this. But this ought to be the icing on the cake, because this is very serious. Him knowing that he can trust me, and that that’s a bond that’s different. That’s another bond within us.”
	PWH01: 46-year-old, Black/African American, female	“What if we fall out with our buddy?”
	PWH10: 61-year-old, White, male	“I think someone who’s conscientious, someone that has an understanding of the importance of what they’re doing, and someone that has a positive relationship with the patient… that there’s not going to be any passive-aggressive interpersonal interactions that might negatively impact the patient.”
	TBY06: 53-year-old, Black/African American, male	“I don’t wanna be like having a heated discussion with my partner and then we gotta do this, you know. Your mental state has to be positive and like even, smooth level.”
	PWH08: 48-year-old, Hispanic/Latino, male	“Is there a possibility that you could switch [TBYs], or would they have to go to the doctor?... Like, let’s say we get into an argument or something and there’s no communication.”

### Innovation

When implementing home-based LAI-ART, PWH and TBYs identified several anticipated facilitators (Table 3) and barriers (Table 4) aligned with the innovation domain. Facilitators included [1] convenience and access, [2] comprehensive training, education, and ongoing support, [3] protecting privacy and reducing stigma, [4] comforting and emotionally supportive care, and [5] fostering autonomy and mutual empowerment. Barriers highlighted concerns around [1] proper injection technique and administration, [2] needlestick injury risk and associated safety concerns for TBYs, [3] ensuring appropriate medication storage and handling, and [4] maintaining timely and reliable injection schedules.

#### Facilitator: convenience and access.

Home-based LAI-ART was widely viewed as a more convenient and accessible alternative to clinic-based care. Participants highlighted its potential to reduce transportation time, address scheduling challenges, alleviate parking and travel costs, and minimize time away from work or caregiving. Home-based LAI-ART was viewed as a way to ease physical and financial strain for many PWH, particularly those with mobility limitations, chronic health conditions, or long commutes. Reported travel times to clinics ranged from 15 minutes to over 2 hours each way, underscoring the burden of clinic visits.

One PWH (PWH01, Table 3) described difficulty attending clinic visits due to health challenges. Another (PWH02) noted the toll of commutes to the clinic exceeding 2 hours each way. Others (PWH03) emphasized the simplicity of delivering medication directly to their home. TBYs echoed these advantages. One individual (TBY02), a healthcare provider, described how reduced travel to the clinic would allow the PWH to remain home and care for their elderly mother-in-law. Another TBY (TBY06) noted that home-based injections would reduce the stress and cost of getting to the clinic. However, one PWH (PWH04) pointed out that home administration could be less convenient in certain circumstances, such as when the clinic is nearby or when the TBY would need to travel to the PWH’s home at a specific time for every injection, potentially creating an additional burden for the TBY.

#### Facilitator: comprehensive training, education, and ongoing support.

Both PWH and TBYs emphasized the need for hands-on, easy-to-understand, and tailored training for laypersons. They wanted training to cover not only injection technique but also anatomical landmarks, maintaining a clean and safe environment, and recognizing and managing potential side effects. Training was viewed as something that should be ongoing rather than a one-time event.

Some PWH noted that their comfort with home injections depended largely on their TBY being well-trained. One PWH (PWH05) expressed confidence that the training process would adequately prepare their TBY, while another (PWH06) felt reassured by having a known, trusted person involved in their care, provided that person received thorough instruction. Concerns about the risks of incorrect administration reinforced the need for clear and precise guidance (PWH06).

TBYs (TBY04) echoed these views, linking their willingness to administer injections to the quality and depth of the training. For some (TBY05), proper instructions would help overcome fears of making a mistake. Others (TBY04) stressed that training should not be limited to technical skills but also include reassurance and ongoing support throughout the process.

#### Facilitator: protecting privacy and reducing stigma.

Participants described home-based LAI-ART as a way to protect confidentiality and reduce the stigma sometimes associated with HIV-specific clinic visits. Receiving injections at home was viewed as a means of safeguarding confidentiality, minimizing the risk of inadvertent disclosure, and reducing the anxiety of being seen in HIV-specific clinical settings.

Some PWH (PWH02) expressed relief at avoiding the visibility of clinic spaces. Others (PWH07) elaborated on concerns about encountering people from their community in the clinic and not knowing what might be said afterward. Some (PWH08) valued home-based LAI-ART because it would eliminate the need to explain their visits to others. PWH also emphasized that discreet medication packaging and delivery for potential home-based injections were key to preserving privacy.

#### Facilitator: comforting and emotionally supportive care.

Participants viewed home-based LAI-ART as a more personalized approach to HIV care. Some PWH (PWH05) valued receiving care in a familiar and private setting, while others (PWH09) highlighted the emotional ease and sense of control that could come from receiving care in their own space with a trusted person. Even those with strong provider relationships (PWH10) saw the appeal, viewing home-based administration as compatible with a philosophy of patient comfort.

TBYs (TBY08) echoed these perspectives, emphasizing the calming nature of the home environment. One TBY with clinical experience (TBY01) contrasted the quiet, privacy, and simplicity of home with the busy, crowded nature of a clinic, underscoring how the home setting could reduce stress and better support PWH’s needs.

#### Facilitator: fostering autonomy and mutual empowerment.

PWH (PWH10) saw home-based LAI-ART as an opportunity to reclaim autonomy, take control of their care, and strengthen their engagement with treatment. This model was also viewed as a way for TBYs to take on a meaningful role, fostering shared growth and connection. One PWH (PWH10) viewed the TBY role as empowering, offering both practical skills-building and emotional fulfillment, while another (PWH07) emphasized the mutual benefit of learning together.

TBYs (TBY09) echoed this sense of empowerment, viewing the experience as an opportunity to gain new skills and contribute meaningfully to their loved one’s health and well-being. Some TBYs, particularly those with healthcare backgrounds (TBY02), saw providing LAIs at home as a natural extension of their professional identity, while others (TBY07) viewed it as a pathway to future roles or opportunities to support others.

#### Barrier: Proper injection technique and administration.

Both PWH and TBYs expressed anxiety about administering injections correctly, including locating the correct injection site, drawing up the medication, and delivering the medication at the appropriate speed. They emphasized the need for precise, step-by-step instructions, hands-on practice, and ongoing support to build confidence and prevent errors.

One PWH (PWH04) highlighted the risks of incorrect technique, recalling a painful experience from a poorly placed injection. TBYs noted the need for clear, detailed guidance (TBY11) and described a strong sense of responsibility and commitment to getting it right (TBY06).

#### Barrier: Needlestick injury risk and associated safety concerns for TBYs.

Accidental needlestick injuries were a concern for both PWH and TBYs. Fears included HIV or hepatitis C transmission, uncertainty about what to do in the event of an incident, and questions about safety protocols and preparedness.

One PWH (PWH10, Table 4) stressed the importance of training and access to emergency resources. TBYs (TBY03) expressed concerns about proper disposal procedures and immediate steps to take following a needlestick injury. Fear of bloodborne infection was also explicitly mentioned (TBY12). For some (TBY08), knowing that the PWH they would be supporting is living with HIV heightened apprehension, despite trust in existing safety protocols.

#### Barrier: Ensuring appropriate medication storage and handling.

Concerns about medication storage, handling, and delivery logistics emerged as a key barrier to home-based LAI-ART. Both PWH and TBYs stressed the importance of maintaining medication integrity, particularly consistent refrigeration and proper preparation.

Participants (PWH08) expressed anxiety about delivery delays or improper storage that could compromise the medication, while others (TBY11) highlighted the need to be present at the time of delivery to ensure safe receipt. At-home storage was another source of concern (TBY07), with participants (PWH10) repeatedly emphasizing that maintaining appropriate temperature is critical to preserving the medication’s effectiveness.

TBYs also expressed concerns about medication preparation, particularly the potential for medication waste when drawing up doses (TBY13). The risk of errors during medication preparation raised questions about whether backup doses would be available in case of accidental mishandling (TBY03).

#### Barrier: Maintaining timely and reliable injection schedules.

The timely administration of injections was considered critical to the success of home-based LAI-ART. Both PWH and TBYs emphasized that delays could compromise treatment effectiveness and lead to clinical consequences. PWH stressed the importance of adhering to the schedule (PWH14) and shared concerns about forgetting (PWH08). Planning and establishing reminders were seen as key strategies to address these challenges. One TBY (TBY11) highlighted the importance of mapping out the entire treatment schedule for the year in advance. While some found it reassuring to have a scheduling window, others (TBY10) emphasized the need to stay close to the target date.

### Inner setting

Within the inner setting domain of home-based LAI-ART, ongoing communication and follow-up emerged as a facilitator, while medication delivery was identified as both a facilitator and a barrier. Anticipated barriers included concerns about [1] access to essential injection supplies and safe disposal options and [2] preparing a safe and distraction-free home injection environment.

#### Facilitator: ongoing communication and follow-up.

Consistent communication with program staff, particularly the clinician trainer, was seen as essential for building and maintaining confidence in home-based LAI-ART. PWHs (PWH05, Table 3) emphasized that regular check-ins, especially after the first few injections, would help reinforce skills and address uncertainties. They valued having open, accessible channels for reaching out with questions or concerns (PWH06), whether through phone calls, video chats, or other real-time support options.

Some (PWH03) suggested that proactive reminders and outreach between injections could help sustain confidence and readiness, particularly given the long gap between doses. Timely reminders were viewed as a way to prevent missed or delayed injections and to maintain momentum. Some (TBY11) also highlighted the usefulness of digital tools, such as their online patient portal, for staying connected with clinic providers, confirming completion of injections, and documenting each step of the process.

#### Facilitator and barrier: medication delivery.

Medication delivery was viewed as both a convenience and a potential obstacle to home-based LAI-ART. For some (PWH15, Table 5), prior experience receiving other medications at home made the idea of the transition feel straightforward and manageable.

Tracking information and delivery updates were considered essential for ensuring that medications arrived safely and on time (TBY03). However, concerns emerged about shipment delays, unclear pickup procedures, and the narrow injection administration window (PWH03). Lost or stolen packages were another worry, particularly for those in multi-unit housing (PWH12), along with the overall complexity of coordinating delivery. Unpredictable delivery times added to the burden (PWH12), and for participants with demanding work schedules, the idea of rigid delivery logistics could create access challenges (PWH03).

#### Barrier: access to essential injection supplies and safe disposal options.

Participants expressed concerns about whether they would receive all the materials needed to support safe and proper injection practices at home. Some wondered if items such as alcohol pads, gloves, and sharps containers would be provided. Safe disposal of used needles was another worry, with uncertainty about available community options for sharps disposal (PWH10). Safety within the home was also noted as a concern (TBY02), underscoring the need for clear guidance and reliable access to injection supplies to ensure safe and timely disposal after each use.

#### Barrier: preparing a safe and distraction-free home injection environment.

Participants emphasized the importance of having a calm, clean, and functional space for administering injections at home, noting concerns about potential disruptions from pets or children, physical setup, and overall safety. Some participants (PWH06) described the need to remove pets from the room to prevent disruptions during injections. The home’s physical layout was also a consideration, with 1 participant (PWH06) pointing out that low furniture could make positioning difficult, and another (PWH04) stressing the importance of preventing strain or injury to the person administering the injection. Cleanliness and organization were also viewed as critical for safe administration (TBY12).

### Outer setting

Laboratory monitoring and coordination emerged as both a facilitator and a barrier within the outer setting domain of home-based LAI-ART. Additional barriers included [1] insurance coverage and cost considerations, and [2] potential loss of clinic connection and routine.

#### Facilitator and barrier: laboratory monitoring and coordination.

Participants emphasized the importance of reliable laboratory monitoring to ensure the success of home-based LAI-ART. While regular laboratory work was seen as reassuring and confidence-building, questions emerged regarding coordination, frequency, and convenience as potential challenges.

Some PWH (PWH04, Table 5) noted that because routine HIV laboratory monitoring still requires in-clinic visits, the overall convenience of home-based LAI-ART could be reduced, as they would still need to travel to the clinic for laboratory work. Others (PWH04) viewed laboratory results as essential for confirming that treatment was effective and correctly administered, suggesting that more frequent monitoring might be needed for the at-home injections. TBYs (TBY12) echoed this need for confirmation to ensure injections were administered correctly.

#### Barrier: insurance coverage and cost considerations.

Uncertainty around insurance coverage and potential out-of-pocket costs emerged as a barrier to participating in home-based LAI-ART. While some participants had confidence in their existing insurance plans and assumed that home administration would be covered in the same way as clinic-based injections, some voiced concerns about how coverage would apply outside of the clinic setting. Some participants (PWH11) who benefited from special funding questioned whether grants that covered clinic-based care would extend to home administration. Another (PWH09) stated that having any out-of-pocket expense would be a hardship.

#### Barrier: potential loss of clinic connection and routine.

Some PWH expressed concern that transitioning to home-based LAI-ART could reduce valued connections with clinic staff and disrupt established routines. For some, clinic visits were not only for medical care but also provided social interaction, familiar faces, and a structured reason to leave the house (PWH01). The shift to home-based LAI-ART raised concerns about modifying these aspects of care. Clinic visits also served practical purposes, allowing participants to address multiple health needs in a single trip (PWH12). The shift to home-based injections raised concerns about losing these benefits, potentially affecting both social engagement and continuity of care.

### Characteristics of individuals

Facilitators and barriers related to individual characteristics encompassed several interrelated factors, including [1] TBY selection considerations and [2] relationship dynamics that support or challenge care. Barriers related to individual characteristics included injection anxiety and emotional readiness.

#### Facilitator and barrier: TBY selection considerations.

Selecting the right TBY was widely viewed as critical to the success of home-based LAI-ART. While practical factors, such as availability and proximity, mattered, participants emphasized that trust, stability, and emotional readiness were central to their decision.

Dependability and a clear understanding of the responsibility involved were recurring themes. Some PWH stressed that a TBY should be reliable and free from substance use during administration (PWH05, Table 5), while others emphasized the need to fully understand the gravity of the role and its potential implications (PWH10).

Relational continuity was also critical (PWH10). Participants valued long-standing, stable relationships (PWH14) and underscored that it was not only the length of the relationship but also confidence that the person would remain in their life over time (PWH16). Physical proximity and frequent contact were also important considerations (PWH13), as was feeling physically comfortable with the person, given the intimate nature of the injections (PWH08).

TBYs (TBY03) expressed uncertainty about managing unexpected disruptions, such as travel or emergencies, and how these might affect adherence.

#### Facilitator and barrier: relationship dynamics that support or challenge care.

Shared responsibility for treatment was often seen as an opportunity to deepen the connection between PWH and TBYs. Some PWH anticipated that supporting one another would build trust and closeness (PWH06, Table 5), and some TBYs emphasized that strengthened bonds could come from taking on this role (TBY07).

At the same time, participants acknowledged the potential for emotional strain. One PWH (PWH01) raised the question of what might happen if they had a falling out with their buddy, while others (PWH10) voiced concerns about interpersonal conflict, emotional volatility, or strained dynamics. Relationship instability was seen as a risk to treatment continuity. Emotional stability was described as essential to creating a safe care environment (TBY06), and some wondered how disruptions might affect their ability to continue with the same TBY (PWH08).

#### Barrier: injection anxiety and emotional readiness.

Both PWH and TBYs expressed anxiety about home-based injections, particularly fears of causing pain, making mistakes, or triggering adverse reactions. While many were open to the model, discomfort with needles and the emotional responsibility of administering care to a loved one emerged as significant concerns for some.

One PWH (PWH16) described the vulnerability involved in handing over that responsibility. TBYs (TBY09) echoed concerns about unintentionally causing harm to someone they care for, and for some (TBY06), the emotional weight of that possibility was enough to raise doubts about continuing.

Participants (TBY08) also worried about injection technique and possible complications. Sterilization and cleanliness were also sources of anxiety, particularly for those without a medical background (TBY12). Needle phobia and general discomfort with injections added another layer of apprehension (PWH12), including challenges in finding someone both willing and emotionally equipped to administer injections (PWH12). Some TBYs (TBY06) questioned their emotional readiness. For others (TBY05), the fear centered on overcoming the first injection experience.

### Implementation process

No facilitators or barriers emerged within the implementation process domain of home-based LAI-ART.

## Discussion

This study is the first to document the perspectives of both PWH and layperson TBYs on home administration of LAI-ART in the United States. These formative findings provide critical insights into the feasibility, acceptability, and support needs of this novel delivery model at a moment when LAI-ART is rapidly transforming HIV care.

Participants expressed a strong interest in home-based administration, citing reduced travel burdens, greater flexibility, increased privacy, and enhanced comfort compared to clinic-based models. Such preferences align with prior research showing that decentralized treatment options can strengthen autonomy, reduce stigma, and expand access to person-centered care [4–7,9,11]. Notably, several participants highlighted that receiving LAI-ART at home from a trusted person could feel less stigmatizing and more empowering than clinic-based options, echoing prior work on the role of trusted non-clinician support [4,23,27].

Home-based models for injectable therapy in other health fields provide important lessons for adapting similar strategies to HIV care. For instance, contraceptive self-injection has been shown to enhance convenience, autonomy, and adherence while reducing logistical barriers such as transportation and clinic scheduling, with positive feedback from both patients and providers [35,36]. Similarly, home-based delivery of injectable treatments for multidrug-resistant tuberculosis has demonstrated feasibility, safety, and cost-effectiveness when coupled with appropriate training and oversight [37,38]. Together, these examples suggest that, with adequate safeguards, the home setting can support safe and effective injection delivery while broadening access and patient agency.

Emerging research indicates that these principles may extend to HIV care. Whitman-Walker Health’s Mobile Outreach Retention and Engagement (MORE) program, for example, found that home administration of cabotegravir/rilpivirine (CAB/RPV) by clinical providers was feasible and linked to high viral suppression rates among individuals with adherence challenges [39]. Additional studies show that people with HIV often prefer options that reduce the frequency of clinic visits, including home-based injection [4,6,24]. Building on this evidence, home-based LAI-ART could reduce structural barriers while enhancing privacy, convenience, and engagement in care. Determining which populations are most likely to benefit will be a key focus of the larger INVITE-Home trial.

Equally important, our data highlight the central role of the PWH-TBY relationship in shaping the feasibility of this approach. Participants prioritized trust, reliability, and emotional closeness when selecting a TBY, suggesting that the quality of this relationship is foundational to the model’s success. Feasibility depended not only on the relationship itself but also on the process of TBY selection—choosing someone with consistency, commitment, and emotional readiness was seen as central, while unresolved conflict or unpredictability risked undermining care. TBY selection was not only a logistical decision but also one shaped by relationship dynamics, with the potential to either strengthen connections or create tension. For some, the model fostered autonomy and mutual empowerment, offering PWH greater self-efficacy and TBYs a meaningful role that blended skill development, emotional connection, and contribution to community care. At the same time, participants noted challenges such as injection anxiety, fear of mistakes, and the psychological toll of caregiving, underscoring the need for training that extends beyond technical skills to address emotional readiness and normalize fear.

Training and ongoing support were described as essential. Participants stressed that lasting confidence in home administration requires preparation that is thorough, clear, reinforced over time, and both technically and emotionally supportive. Specific concerns included mastering injection technique, avoiding needlestick injuries, and ensuring clear post-exposure protocols and access to emergency resources such as PEP. These reflections highlight the importance of training systems that address both safety and confidence.

Participants also emphasized that strong coordination with healthcare teams remains essential. Reliable medication delivery, clear storage and disposal systems, and contingency planning were described as essential for safe home use. Medication delivery itself was a double-edged sword—highly convenient for some but a potential barrier for others if systems were inconsistent or unclear. Participants also emphasized the need for injection supplies, safe disposal options, and clear guidance on creating a distraction-free environment. Ongoing communication and follow-up with clinical teams were described as vital for maintaining skills, confidence, and continuity of care. Similarly, while laboratory monitoring could strengthen trust in home-based LAI-ART, it also introduced logistical challenges, highlighting the need for flexible and well-coordinated monitoring plans. Structural barriers, including insurance coverage, billing processes, and cost considerations, reinforced the need for upfront communication and proactive benefits navigation to support equitable access. Finally, for some PWH, shifting care to the home raised concerns about losing clinic connections and routines, suggesting that strategies are needed to sustain provider relationships and preserve aspects of clinic-based support.

These findings suggest that home-based LAI-ART with trained TBYs could expand access to more flexible, empowering, and person-centered HIV care in the United States. Realizing this potential will require proactive planning, robust training, and carefully designed support systems that anticipate not only technical and logistical demands but also the emotional and relational dimensions of home-based HIV treatment.

While this formative study focused on participants’ perceptions across 4 CFIR domains, the implementation process will be examined in the next phase of our work. We are currently developing communication tools (e.g., reporting mechanisms between PWH, TBYs, and the clinical team) and quality improvement strategies (e.g., iterative refinements to the injection training) to support delivery of home-based LAI-ART. These efforts will be piloted to further refine implementation in preparation for scaling to a larger trial.

### Limitations

This formative study was conducted across 4 clinics within a single metropolitan area, which may limit the generalizability of findings to regions with different healthcare systems, patient demographics, or resources. While the clinics varied in population focus and care models, the findings may not reflect the full range of experiences nationally. Because our results were based on self-reports, findings may also be influenced by social desirability bias. In addition, although interest in home-based injections was not an eligibility requirement, participants who completed interviews may have been more open to or interested in this approach than individuals who did not participate, potentially skewing perspectives in a favorable direction. Finally, because interviews were conducted before the intervention was implemented, responses reflected participants’ expectations and assumptions rather than their lived experiences. These early insights help inform planning but may differ from what emerges during real-world implementation. Future phases of INVITE-Home will explore implementation in practice and assess how anticipated facilitators and barriers align with actual experiences [28].

## Conclusion

As LAI-ART delivery expands, home-based administration offers a promising option for improving flexibility, privacy, and comfort, particularly for those facing barriers to regular clinic attendance. This formative study highlights that PWH and TBYs find the model acceptable when paired with adequate support, clear protocols, and responsive communication. Successful implementation depends on more than technical training; it requires emotional readiness, trust in the TBY, and confidence in safety and monitoring systems. These findings can inform patient education, TBY training, and systems planning to ensure that home-based LAI-ART is feasible and empowering for those it intends to serve.
